# Comparison of sexual function outcomes after vNOTES and laparoscopic tubal sterilization

**DOI:** 10.3389/fmed.2026.1926568

**Published:** 2026-08-25

**Authors:** Begum Ertan, Gokce Aykanat, Onur Yavuz, Ufuk Atlihan, Ferruh Acet

**Affiliations:** 1 Department of Gynecology and Obstetrics, Saglik Bakanligi Akhisar Mustafa Kirazoglu Devlet Hastanesi, Manisa, Türkiye; 2 Department of Gynecology and Obstetrics, Saglik Bakanligi Kinik Devlet Hastanesi, İzmir, Türkiye; 3 Department of Gynecology and Obstetrics, Dokuz Eylül Üniversitesi, İzmir, Türkiye; 4 Department of Gynecology and Obstetrics, İzmir Demokrasi Üniversitesi, İzmir, Türkiye; 5 Özel Tavmergen IVF Clinic Infertilite ve Tüp Bebek Merkezi, İzmir, Türkiye

**Keywords:** dyspareunia, female sexual function index, sexual function, tubal sterilization, vNOTES

## Abstract

**Objective:**

To compare postoperative sexual function outcomes following vaginal natural orifice transluminal endoscopic surgery (vNOTES) and laparoscopic tubal sterilization (LTS).

**Material and Methods:**

This retrospective comparative cohort analysis included 64 women with prospectively collected routine clinical data who underwent permanent surgical sterilization using vNOTES (*n* = 31) or laparoscopy (*n* = 33). Dyspareunia VAS, QSES, and FSFI scores were assessed preoperatively and at 6 months. Six-month outcomes were compared using ANCOVA adjusted for the corresponding baseline scores. Adjusted mean differences, 95% confidence intervals, and partial eta squared (partial η^2^) were reported.

**Results:**

After adjustment for baseline scores, the 6-month dyspareunia VAS score was significantly higher in the vNOTES group than in the LTS group (AMD, 0.44; 95% CI, 0.22 to 0.66; unadjusted *p* < 0.001; Holm-adjusted *p* < 0.002; partial η^2^ = 0.210). No significant differences were observed in the primary outcome, total FSFI (AMD, -1.00; 95% CI, -2.70 to 0.70; *p* = 0.240), or in QSES (AMD, -0.40; 95% CI, -2.10 to 1.30; Holm-adjusted *p* = 0.640). Among the exploratory FSFI domains, only satisfaction remained significantly lower in the vNOTES group after Holm correction (AMD, -0.39; 95% CI, -0.61 to -0.17; Holm-adjusted *p* < 0.006). The unadjusted difference in lubrication did not remain significant after correction (Holm-adjusted *p* = 0.055).

**Conclusion:**

vNOTES was associated with higher dyspareunia scores and lower satisfaction-domain scores at 6 months; however, no significant between-group differences were observed in total FSFI or QSES scores. These findings should be interpreted cautiously because of the retrospective, nonrandomized design and small sample size.

## Introduction

Tubal sterilization, achieved through occlusion or removal of the fallopian tubes, is a highly effective and widely used method of permanent contraception worldwide (1). Conventional laparoscopy has long been considered the standard minimally invasive approach for tubal sterilization (2). However, the introduction of vaginal natural orifice transluminal endoscopic surgery (vNOTES) has expanded the range of minimally invasive surgical options by providing access to the peritoneal cavity through a transvaginal route without abdominal incisions (3, 4). Available studies have generally reported no significant deterioration in female sexual function after vNOTES; however, the evidence remains limited and heterogeneous, particularly for women undergoing tubal sterilization (5). In recent years, vNOTES has been increasingly used for hysterectomy, adnexal surgery, and sterilization procedures. Among its proposed advantages, improvements in postoperative quality of life and sexual well-being have attracted increasing attention (4). Sexual function is a multidimensional concept influenced by physical, psychological, and social factors. Surgical interventions involving the pelvic organs may alter sexual function through postoperative pain, tissue trauma, anatomic changes, or psychological factors (6). In particular, comparative data on sexual-function outcomes following vNOTES and laparoscopic tubal sterilization (LTS) remain scarce. Moreover, the adoption of vNOTES remains restricted by the need for specialized surgical expertise and equipment, highlighting the importance of high-quality clinical evidence to guide surgical decision-making. Therefore, this study aimed to compare postoperative sexual function outcomes between women undergoing vNOTES and conventional LTS.

## Materials and methods

### Study design and ethical approval

This study was designed as a retrospective comparative cohort analysis of prospectively collected routine clinical data. Sexual-function assessments had been routinely performed at our institution before the present study was planned, and the decision to analyze these data was made after completion of the clinical follow-up period. The study was conducted in accordance with the Declaration of Helsinki. Ethical approval was obtained from the Buca Seyfi Demirsoy Training and Research Hospital Non-Interventional Research Ethics Committee (Decision No. 2026/04-73; April 29, 2026). The surgical procedures and sexual-function assessments were performed between January 2024 and September 2025 as part of routine clinical care and preceded the conception of the present study. The ethics approval obtained on April 29, 2026, specifically covered the retrospective analysis of previously collected clinical and questionnaire data. Because the study involved the retrospective analysis of anonymized routine clinical data, the ethics committee waived the requirement for study-specific informed consent.

### Study population

**FIGURE 1:**
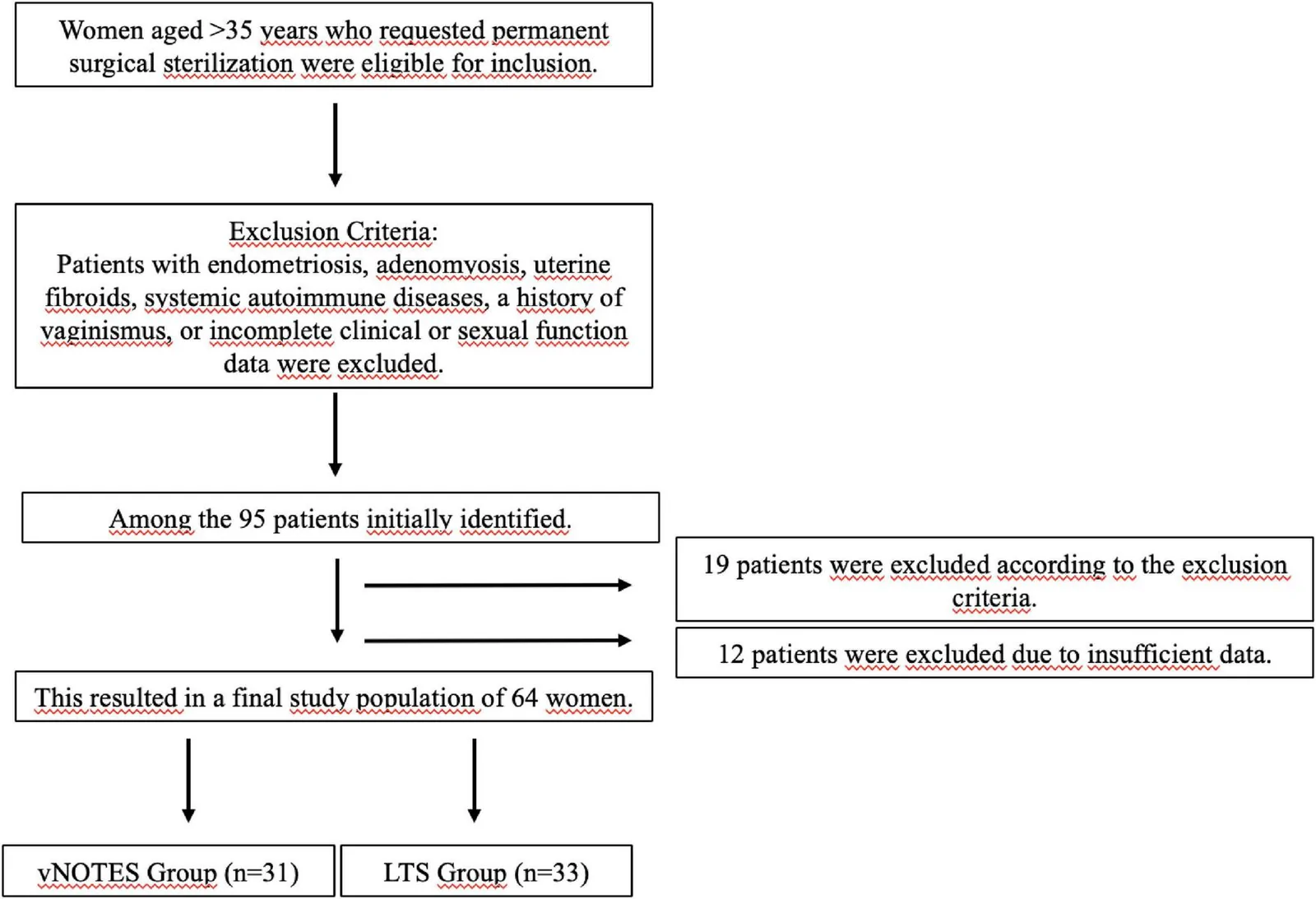
Flowchart of patient selection and group classification. Women aged > 35 years undergoing permanent surgical sterilization were screened and classified according to the surgical approach as vNOTES or LTS.

The medical records of 95 women who underwent bilateral tubal sterilization for permanent contraception between January 2024 and September 2025 were retrospectively reviewed. Women aged > 35 years who requested permanent surgical sterilization were eligible for inclusion. Nineteen women were excluded because of systemic or autoimmune disease (*n* = 5), endometriosis (*n* = 4), uterine fibroids (*n* = 3), adenomyosis (*n* = 3), a history of vaginismus (*n* = 2), or Bartholin gland pathology (*n* = 2). An additional 12 women, who had completed the baseline questionnaires, were excluded because their 6-month questionnaires were unavailable. No imputation was performed, and the analysis was restricted to patients with complete baseline and 6-month outcome data. The remaining 64 women were classified according to the surgical approach as the vNOTES group (*n* = 31) or the laparoscopic tubal sterilization group (*n* = 33) (Figure 1). Because of the retrospective design, no formal *a priori* sample-size calculation was performed. The sample size was determined by the number of patients who underwent the eligible procedures during the predefined study period and had complete baseline and 6-month outcome data. Accordingly, all 64 eligible patients with complete data were included in the analysis.

### Surgical approach and techniques

Because of the retrospective and non-randomized design, the surgical approach was selected through shared decision-making between the patient and the operating surgeon. Selection of vNOTES or conventional laparoscopy was based on patient preference, surgical history, pelvic examination findings, anatomical suitability for transvaginal access, the presence or suspicion of pelvic adhesions, and the surgeon’s clinical assessment and experience. Patients with clinical or anatomical findings considered unfavorable for safe transvaginal access underwent conventional laparoscopy. All procedures were performed by surgeons experienced in vNOTES who had surpassed the initial learning curve. Bilateral partial salpingectomy with tubal coagulation and segmental excision was performed in both groups. All patients received 1 g of intravenous ceftriaxone preoperatively for antibiotic prophylaxis.

In the vNOTES group, patients were placed in the dorsal lithotomy position. After vaginal preparation, an approximately 3-cm posterior colpotomy was performed through the posterior vaginal fornix. An Alexis wound retractor was inserted through the colpotomy to establish transvaginal access. Pneumoperitoneum was established and maintained at an intra-abdominal pressure of 12 mmHg. A segment of each fallopian tube was coagulated and divided using a LigaSure vessel-sealing device. The excised tubal segments were removed through the posterior colpotomy. After hemostasis was confirmed, the Alexis retractor was removed, and the colpotomy incision was closed with a continuous 0 Vicryl suture.

In the laparoscopic group, pneumoperitoneum was established and maintained at an intra-abdominal pressure of 12 mmHg. One 10-mm umbilical trocar and one 5-mm ancillary trocar were inserted. As in the vNOTES group, a segment of each fallopian tube was coagulated and divided using a LigaSure vessel-sealing device. The excised tubal segments were removed through the 5-mm ancillary port. After hemostasis was confirmed, the instruments were removed and the trocar incisions were closed using the standard technique. Postoperative analgesia consisted of oral ibuprofen administered as needed. Women in both groups were advised to avoid sexual intercourse for 6 weeks after surgery. In the vNOTES group, this recommendation was intended to allow adequate healing of the posterior colpotomy incision.

### Perioperative and postoperative outcomes

Perioperative and postoperative outcomes were obtained retrospectively from the medical records. The evaluated outcomes included operative time, estimated blood loss, intraoperative complications, early postoperative pain, length of hospital stay, postoperative infection, hematoma, surgical-site complications, readmission, and reintervention. Vaginal wound and colpotomy-related complications were assessed in the vNOTES group, whereas trocar-site complications were assessed in the laparoscopic group. Readmission and reintervention were evaluated within 30 days after surgery. These outcomes were summarized descriptively because they were included as supplementary safety and recovery measures rather than prespecified study outcomes.

### Outcome assessment and follow-up

Sexual-function outcomes were assessed 1 day before surgery and at the routine 6-month postoperative outpatient visit. All women included in the analysis reported being sexually active during both assessment periods. Dyspareunia was assessed using a 0–10 visual analog scale (VAS) based on the women’s recent sexual intercourse, with 0 indicating no pain and 10 indicating the worst imaginable pain. No fixed recall period was specified. The VAS assessed overall dyspareunia and did not distinguish between superficial and deep dyspareunia.

Female sexual function was assessed using the validated Turkish version of the Female Sexual Function Index (FSFI), a 19-item self-administered questionnaire comprising six domains: desire, arousal, lubrication, orgasm, satisfaction, and pain (7, 8). Higher total and domain scores indicate better sexual function. The quality of sexual experience was evaluated using the validated Turkish version of the Quality of Sexual Experience Scale (QSES), which ranges from 7 to 49, with higher scores indicating a better sexual experience (9, 10).

The questionnaires were administered by the operating surgeon as part of routine clinical follow-up. Patients completed the questionnaires independently in a private setting, without the operating surgeon or accompanying persons being present, and returned the completed forms to the operating surgeon. The 6-month questionnaires were completed during scheduled outpatient visits. Because the assessments were conducted within routine clinical care, the surgeon administering and evaluating the questionnaires was aware of the surgical approach. Therefore, questionnaire administration and outcome assessment were not blinded.

### Statistical analysis

Statistical analyses were performed using IBM SPSS Statistics for Windows, version 26.0 (IBM Corp., Armonk, NY, USA). The distribution of continuous variables was assessed using the Kolmogorov–Smirnov test and by visual inspection of histograms and Q–Q plots. Continuous variables are presented as mean ± standard deviation (SD), and categorical variables as numbers and percentages. Perioperative and postoperative outcomes were summarized descriptively using mean ± SD or median (interquartile range) for continuous variables and number (percentage) for categorical variables. No formal hypothesis testing was performed for these supplementary outcomes. Baseline continuous variables were compared between the vNOTES and laparoscopic tubal sterilization (LTS) groups using the independent-samples *t*-test, while categorical variables were compared using the chi-square test. 6-month sexual function outcomes were compared between the surgical groups using analysis of covariance (ANCOVA). Separate ANCOVA models were constructed for dyspareunia visual analog scale (VAS), Quality of Sexual Experience Scale (QSES), total Female Sexual Function Index (FSFI), and each FSFI domain score. In each model, the 6-month score was entered as the dependent variable, surgical group as the fixed factor, and the corresponding preoperative score as the covariate. The homogeneity of regression slopes assumption was evaluated by testing the interaction between surgical group and the corresponding baseline score. Homogeneity of variance was assessed using Levene’s test, and the normality of model residuals was evaluated using Q–Q plots. Total FSFI score was designated as the primary outcome. Dyspareunia VAS and QSES scores were classified as secondary outcomes, while the six FSFI domain scores were considered exploratory outcomes. To account for multiple testing, the Holm procedure was applied separately to the secondary outcome family and the exploratory FSFI-domain family. No multiplicity adjustment was applied to the single primary outcome. Statistical significance for secondary and exploratory outcomes was determined using the Holm-adjusted *p*-values. Baseline-adjusted between-group effects are reported as adjusted mean differences, calculated as vNOTES minus LTS, with 95% confidence intervals (CIs). A positive adjusted mean difference indicates a higher adjusted 6-month score in the vNOTES group, whereas a negative value indicates a lower adjusted score in the vNOTES group. Partial eta squared (partial η^2^) was reported as the measure of effect size. To enhance the clinical interpretability of the findings, the proportions of women with a total FSFI score of ≤ 26.55 were calculated separately for each group at baseline and 6 months (11). This validated cut-off was interpreted as indicating possible sexual dysfunction rather than establishing a clinical diagnosis. These proportions and their absolute changes over time were evaluated descriptively. All statistical tests were two-sided, and a *p*-value < 0.05 was considered statistically significant.

## Results

A total of 64 women were included in the study, including 31 patients in the vNOTES group and 33 patients in the LTS group. The mean age was comparable between the vNOTES and LTS groups (38.1 ± 1.4 vs. 37.9 ± 1.3 years, respectively; *p* = 0.56). No significant between-group differences were observed in body mass index (24.2 ± 1.9 vs. 24.1 ± 1.7 kg/m^2^; *p* = 0.83), gravidity (2.2 ± 1.4 vs. 2.3 ± 1.3; *p* = 0.77), or parity (1.8 ± 0.9 vs. 1.9 ± 0.8; *p* = 0.64). The distribution of delivery mode was also similar between the groups, with cesarean delivery rates of 22.6% (7/31) in the vNOTES group and 24.2% (8/33) in the LTS group (*p* = 0.82) (Table 1). No material violations of the normality of residuals, homogeneity of variance, or homogeneity of regression slopes assumptions were identified.

**TABLE 1:** Demographic characteristics of the groups.

Characteristics	vNOTES group (*n* = 31)	LTS group (*n* = 33)	*P*-value
Age	38.1 ± 1.4	37.9 ± 1.3	0.56
BMI (kg/m^2^)	24.2 ± 1.9	24.1 ± 1.7	0.83
Gravidity	2.2 ± 1.4	2.3 ± 1.3	0.77
Parity	1.8 ± 0.9	1.9 ± 0.8	0.64
Mode of delivery			0.82
*C/S*	22.6% (7/31)	24.2% (8/33)	
*NSVD*	77.4% (24/31)	75.8% (25/33)	

BMI, body mass index; C/S, Caesarean section; NSVD, normal spontaneous vaginal delivery.

Sexual function outcomes at baseline and at 6 months, together with the baseline-adjusted between-group comparisons, are presented in Table 2. Total FSFI score was designated as the primary outcome. Total FSFI scores decreased from 28.8 ± 3.8 at baseline to 27.4 ± 3.5 at 6 months in the vNOTES group and from 28.5 ± 3.7 to 28.2 ± 3.5 in the LTS group. After adjustment for baseline total FSFI scores, no significant difference was observed between the groups at 6 months (adjusted mean difference [AMD], -1.00; 95% confidence interval [CI], -2.70 to 0.70; *p* = 0.240; partial η^2^ = 0.023).

**TABLE 2:** Baseline and 6-month sexual function outcomes and baseline-adjusted comparisons between the surgical groups.

Outcome	vNOTES baseline, mean ± SD	vNOTES 6 months, mean ± SD	LTS baseline, mean ± SD	LTS 6 months, mean ± SD	Adjusted mean difference^1^	95% CI^1^	Unadjusted *p*	Holm-adjusted *p*	Partial η^21^
Dyspareunia VAS	3.3 ± 0.5	3.8 ± 0.4	3.8 ± 0.5	3.4 ± 0.5	0.44	0.22 to 0.66	<0.001	<0.002	0.210
QSES	38.1 ± 3.2	37.5 ± 3.5	38.3 ± 3.5	38.1 ± 3.3	-0.40	-2.10 to 1.30	0.640	0.640	0.004
Total FSFI	28.8 ± 3.8	27.4 ± 3.5	28.5 ± 3.7	28.2 ± 3.5	-1.00	-2.70 to 0.70	0.240	NA	0.023
Desire	5.1 ± 0.6	4.9 ± 0.5	5.1 ± 0.7	4.9 ± 0.4	0.02	-0.20 to 0.24	0.860	1.000	0.001
Arousal	4.9 ± 0.4	4.8 ± 0.5	4.8 ± 0.3	4.7 ± 0.4	0.08	-0.14 to 0.30	0.470	1.000	0.008
Lubrication	4.7 ± 0.4	4.4 ± 0.5	4.7 ± 0.6	4.7 ± 0.4	-0.29	-0.51 to -0.07	0.011	0.055	0.105
Orgasm	4.5 ± 0.5	4.3 ± 0.4	4.5 ± 0.4	4.3 ± 0.6	-0.01	-0.25 to 0.23	0.930	1.000	<0.001
Satisfaction	4.6 ± 0.5	4.3 ± 0.5	4.6 ± 0.6	4.7 ± 0.4	-0.39	-0.61 to -0.17	<0.001	<0.006	0.190
Pain	5.1 ± 0.3	4.6 ± 0.5	5.1 ± 0.5	4.8 ± 0.3	-0.19	-0.39 to 0.01	0.061	0.244	0.057

Values are presented as mean ± standard deviation. Adjusted mean differences represent vNOTES minus LTS and were estimated using analysis of covariance, with the corresponding baseline score included as a covariate. Total FSFI was designated as the primary outcome. Dyspareunia VAS and QSES were classified as secondary outcomes, and the FSFI domains were considered exploratory. Holm correction was applied separately to the secondary outcome family and the exploratory FSFI-domain family. Holm adjustment was not applicable to the single primary outcome. CI, confidence interval; FSFI, female sexual function index; LTS, laparoscopic tubal sterilization; QSES, quality of sexual experience scale; SD, standard deviation; VAS, visual analog scale.

Dyspareunia VAS and QSES were evaluated as secondary outcomes. Dyspareunia VAS scores increased from 3.3 ± 0.5 at baseline to 3.8 ± 0.4 at 6 months in the vNOTES group, whereas they decreased from 3.8 ± 0.5 to 3.4 ± 0.5 in the LTS group. After adjustment for baseline VAS scores, the 6-month VAS score was significantly higher in the vNOTES group than in the LTS group (AMD, 0.44; 95% CI, 0.22 to 0.66; unadjusted *p* < 0.001; Holm-adjusted *p* < 0.002; partial η^2^ = 0.210). QSES scores decreased from 38.1 ± 3.2 to 37.5 ± 3.5 in the vNOTES group and from 38.3 ± 3.5 to 38.1 ± 3.3 in the LTS group. No significant baseline-adjusted difference was observed in QSES scores (AMD, -0.40; 95% CI, -2.10 to 1.30; unadjusted *p* = 0.640; Holm-adjusted *p* = 0.640; partial η^2^ = 0.004).

The six FSFI domain scores were evaluated as exploratory outcomes. Satisfaction scores were significantly lower in the vNOTES group than in the LTS group and remained significant after correction for multiple testing (AMD, -0.39; 95% CI, -0.61 to -0.17; unadjusted *p* < 0.001; Holm-adjusted *p* < 0.006; partial η^2^ = 0.190). Lubrication scores were lower in the vNOTES group in the unadjusted analysis (AMD, -0.29; 95% CI, -0.51 to -0.07; unadjusted *p* = 0.011), but the difference did not remain statistically significant after Holm correction (Holm-adjusted *p* = 0.055; partial η^2^ = 0.105). No significant multiplicity-adjusted between-group differences were observed in desire (Holm-adjusted *p* = 1.000), arousal (Holm-adjusted *p* = 1.000), orgasm (Holm-adjusted *p* = 1.000), or pain scores (Holm-adjusted *p* = 0.244) (Table 2).

Based on the validated FSFI cut-off of ≤ 26.55, 17 of 31 women (54.8%) in the vNOTES group and 19 of 33 women (57.6%) in the laparoscopic group screened positive for possible sexual dysfunction preoperatively. At 6 months, the corresponding proportions were 14 of 31 (45.2%) and 20 of 33 (60.6%), respectively. This represented an absolute decrease of 9.6 percentage points in the vNOTES group and an increase of 3.0 percentage points in the laparoscopic group. These findings were evaluated descriptively, as the FSFI cut-off represents a screening threshold rather than a clinical diagnosis (Table 3).

**TABLE 3:** Proportion of women meeting the FSFI screening threshold for possible sexual dysfunction at baseline and 6 months.

Assessment time	vNOTES group (*n* = 31), *n* (%)	Laparoscopic group (*n* = 33), *n* (%)
Preoperative	17 (54.8)	19 (57.6)
Six months postoperative	14 (45.2)	20 (60.6)
Absolute change, percentage points	-9.6	+3.0

FSFI, female sexual function index. A total FSFI score of ≤ 26.55 was considered indicative of possible sexual dysfunction. This threshold represents a screening criterion and not a clinical diagnosis. Negative percentage-point changes indicate a reduction in the proportion of women screening positive for possible sexual dysfunction.

The mean operative time was 35.5 ± 8.2 min in the vNOTES group and 41.8 ± 9.5 min in the laparoscopic group. The corresponding estimated blood loss values were 28.6 ± 12.4 and 31.1 ± 14.7 mL, respectively. At 6 h postoperatively, mean pain VAS scores were 2.8 ± 0.9 in the vNOTES group and 3.1 ± 1.0 in the laparoscopic group, while the mean lengths of hospital stay were 18.4 ± 4.2 and 19.2 ± 4.6 h, respectively. One intraoperative complication (3.0%) occurred in the laparoscopic group. One vaginal wound complication (3.2%) was recorded in the vNOTES group, and one trocar-site complication (3.0%) was recorded in the laparoscopic group. No postoperative infection, hematoma, readmission, or reintervention was recorded in either group (Table 4).

**TABLE 4:** Perioperative and postoperative outcomes according to surgical approach.

Outcome	vNOTES group (*n* = 31)	Laparoscopic group (*n* = 33)
Operative time, min	35.5 ± 8.2	41.8 ± 9.5
Estimated blood loss, mL	28.6 ± 12.4	31.1 ± 14.7
Postoperative pain VAS at 6 h	2.8 ± 0.9	3.1 ± 1.0
Length of hospital stay, h	18.4 ± 4.2	19.2 ± 4.6
Intraoperative complications	0 (0)	1 (3.0)
Vaginal wound complications	1 (3.2)	Not applicable
Trocar-site complications	Not applicable	1 (3.0)
Postoperative infection	0 (0)	0 (0)
Hematoma	0 (0)	0 (0)
Readmission within 30 days	0 (0)	0 (0)
Reintervention within 30 days	0 (0)	0 (0)

Values are presented as mean ± standard deviation or number (percentage), as appropriate. VAS, visual analog scale.

## Discussion

The present study compared changes in sexual-function outcomes following vNOTES and laparoscopic tubal sterilization. After adjustment for the corresponding baseline scores, no significant between-group difference was observed in total FSFI, the designated primary outcome, or in QSES at 6 months. However, dyspareunia VAS scores were significantly higher in the vNOTES group. Among the exploratory FSFI domains, only satisfaction remained significantly lower in the vNOTES group after correction for multiple testing; the differences in lubrication and pain did not remain statistically significant after Holm adjustment. These findings suggest that vNOTES may be associated with differences in certain aspects of postoperative sexual well-being, without demonstrating a significant difference in overall sexual function or quality of sexual experience.

Evidence regarding sexual function after vNOTES remains limited and heterogeneous. In a systematic review including 28 gynecologic and nongynecologic studies, Krull et al. reported that vNOTES generally did not significantly impair female sexual function, except in a limited number of specific clinical settings (12). Similarly, Genco et al., in a comparison of vNOTES and laparoscopic tubal sterilization, found comparable postoperative sexual-function outcomes between the two approaches, while vNOTES was associated with lower early postoperative and shoulder pain (13). The higher adjusted dyspareunia VAS score observed in our study differs from these findings. Differences in patient selection, surgical technique, outcome instruments, follow-up duration, and baseline adjustment may partly account for the inconsistency between studies. Notably, our analysis directly compared baseline-adjusted 6-month outcomes rather than inferring a treatment difference from separate within-group changes.

Studies involving other gynecologic indications have also generally reported preservation of sexual function after vNOTES. Housmans et al. found that vNOTES hysterectomy provided surgical outcomes comparable to conventional laparoscopic hysterectomy, with potential perioperative advantages in appropriately selected patients (14). Berisha et al. reported a low risk of persistent sexual dysfunction following vNOTES for benign adnexal procedures, although transient deep dyspareunia occurred in a small proportion of women (15). Xu et al. similarly found no significant adverse effect of vNOTES on postoperative female sexual function in uterus-conserving procedures (16). Randomized and meta-analytic evidence has also demonstrated favorable perioperative and quality-of-life outcomes after vNOTES without clear evidence of deterioration in sexual function (17, 18). Differences between these reports and the present findings may reflect variation in surgical indication and procedure. Tubal sterilization is performed in otherwise healthy women for permanent contraception, and expectations regarding postoperative recovery and sexual well-being may differ from those of women undergoing surgery for symptomatic gynecologic disease.

The mechanism underlying the higher dyspareunia VAS score observed after vNOTES cannot be determined from the present data. Posterior colpotomy and subsequent vaginal healing could theoretically be associated with temporary tissue sensitivity, local discomfort, or concern regarding resumption of intercourse. Psychological responses to the transvaginal access route may also influence pain perception and sexual experience. However, these explanations remain hypothetical because vaginal sensitivity, scar characteristics, psychological factors, and the actual timing of resumption of intercourse were not systematically assessed. Moreover, the FSFI pain-domain difference was not statistically significant after multiplicity correction. The VAS and FSFI pain domain should not be regarded as interchangeable because they use different scoring structures and assess pain within different measurement contexts.

The statistical significance of the dyspareunia result should also be distinguished from its clinical significance. The adjusted between-group difference was 0.44 points on a 0–10 VAS, with a 95% confidence interval of 0.22 to 0.66. Although statistically significant, this absolute difference was small, and its clinical importance remains uncertain because an accepted minimal clinically important difference specific to dyspareunia after tubal sterilization was not available. Likewise, the adjusted differences in total FSFI and QSES were small and their confidence intervals included zero. The satisfaction-domain difference was also modest and originated from an exploratory analysis, despite remaining significant after Holm correction. Therefore, it should be interpreted as hypothesis-generating rather than definitive evidence of a clinically important between-group difference.

The descriptive FSFI threshold analysis provides additional clinical context but does not establish superiority of either approach. The proportion of women with an FSFI score of ≤ 26.55 decreased from 54.8 to 45.2% in the vNOTES group and increased from 57.6 to 60.6% in the laparoscopic group. These proportions were not subjected to a prespecified inferential comparison, and the threshold indicates possible sexual dysfunction rather than a clinical diagnosis. Consequently, these descriptive changes should not be interpreted as evidence that one surgical approach improved or worsened sexual function. Their lack of a clear parallel with the adjusted continuous-score findings further supports cautious interpretation of the clinical relevance of the observed differences.

The supplementary perioperative findings showed relatively low blood loss, short hospital stays, and few complications in both groups. However, these outcomes were summarized descriptively and the study was not designed or powered to establish perioperative superiority. Similarly, cosmetic satisfaction was not directly measured and no conclusion regarding cosmetic benefit can be drawn from the present data. Surgical experience should also be considered when interpreting vNOTES outcomes because the technique requires familiarity with transvaginal pelvic access and has a recognized learning curve (19). In this study, the procedures were performed by surgeons who had surpassed the initial learning curve, although operator-related effects cannot be completely excluded.

The principal strengths of this study include the comparison of two surgical approaches used for the same indication, the availability of preoperative assessments, the use of validated Turkish versions of the FSFI and QSES, and the evaluation of complementary patient-reported outcomes. The baseline-adjusted analysis directly addressed whether 6-month outcomes differed between the surgical groups, while reporting adjusted mean differences, confidence intervals, and effect sizes. Correction for multiple testing also reduced the risk of false-positive interpretation of the exploratory domain analyses.

Several limitations should nevertheless be acknowledged. The retrospective, nonrandomized design introduces the possibility of selection bias and confounding by indication because the surgical approach was influenced by patient preference, surgical history, anatomical suitability, suspected adhesions, and surgeon judgment. Potentially relevant variables–including previous pelvic surgery, chronic pelvic pain, menopausal status, hormonal treatment, relationship status, psychological conditions, psychotropic medication use, and the actual timing of resumption of intercourse–were not consistently available and could not be included in a multivariable or propensity-based analysis. Although the ANCOVA models adjusted for the corresponding baseline scores, residual confounding remains possible and the observed associations should not be interpreted as causal effects.

No formal *a priori* sample-size calculation was performed, and the relatively small sample limited the precision of the estimates, particularly for exploratory FSFI domains. Accordingly, nonsignificant results should not be interpreted as demonstrating equivalence between the approaches. The single-center setting may also limit generalizability. Sexual outcomes were self-reported, and the questionnaires were administered within routine care by the operating surgeon, who was aware of the surgical approach, potentially introducing response or assessment bias. The dyspareunia VAS did not use a fixed recall period and did not distinguish between superficial and deep dyspareunia. Exclusion of women without complete 6-month questionnaires may have introduced attrition bias. Finally, follow-up was limited to 6 months and does not clarify whether the observed dyspareunia difference persisted, diminished, or increased over time. Larger prospective studies with standardized pain assessment, blinded outcome evaluation where feasible, longer follow-up, and measurement of relevant psychological, relational, and clinical confounders are needed.

## Conclusion

In this retrospective comparative cohort study, no significant baseline-adjusted between-group difference was observed in total FSFI, the primary outcome, or in QSES at 6 months. vNOTES was associated with a higher dyspareunia VAS score and a lower exploratory FSFI satisfaction-domain score compared with laparoscopic tubal sterilization. However, the absolute differences were small and their clinical significance remains uncertain. Given the nonrandomized design, small sample size, potential residual confounding, and limited follow-up, these findings should be considered hypothesis-generating rather than evidence of a causal effect. Larger prospective studies are required to confirm these associations and determine their clinical relevance.

## Data Availability

The raw data supporting the conclusions of this article will be made available by the authors, without undue reservation.
